# Hydrogen Production in Microbial Electrolysis Cells Using an Alginate Hydrogel Bioanode Encapsulated with a Filter Bag

**DOI:** 10.3390/polym16141996

**Published:** 2024-07-12

**Authors:** Lea Ouaknin Hirsch, Bharath Gandu, Abhishiktha Chiliveru, Irina Amar Dubrovin, Avinash Jukanti, Alex Schechter, Rivka Cahan

**Affiliations:** 1 Department of Chemical Engineering, Ariel University, Ariel 40700, Israel; lea.ouaknin@gmail.com (L.O.H.); bharathgandu@gmail.com (B.G.); abhishikthachilivery@gmail.com (A.C.); irinadubrovin@gmail.com (I.A.D.); javinash1007@gmail.com (A.J.); 2 Department of Environmental Studies, University of Delhi, New Delhi 110007, India; 3 Department of Chemical Sciences, Ariel University, Ariel 40700, Israel; salex@ariel.ac.il; 4 Research and Development Centre for Renewable Energy, New Technologies, Research Centre (NTC), University of West Bohemia, 30100 Pilsen, Czech Republic

**Keywords:** microbial electrolysis cell, alginate hydrogel, impedance, hydrogen evolution reaction

## Abstract

The bacterial anode of microbial electrolysis cells (MECs) is the limiting factor in a high hydrogen evolution reaction (HER). This study focused on improving biofilm attachment to a carbon-cloth anode using an alginate hydrogel. In addition, the modified bioanode was encapsulated by a filter bag that served as a physical barrier, to overcome its low mechanical strength and alginate degradation by certain bacterial species in wastewater. The MEC based on an encapsulated alginate bioanode (alginate bioanode encapsulated by a filter bag) was compared with three controls: an MEC based on a bare bioanode (non-immobilized bioanode), an alginate bioanode, and an encapsulated bioanode (bioanode encapsulated by a filter bag). At the beginning of the operation, the Rct value for the encapsulated alginate bioanode was 240.2 Ω, which decreased over time and dropped to 9.8 Ω after three weeks of operation when the *Geobacter* medium was used as the carbon source. When the MECs were fed with wastewater, the encapsulated alginate bioanode led to the highest current density of 9.21 ± 0.16 A·m^−2^ (at 0.4 V), which was 20%, 95%, and 180% higher, compared to the alginate bioanode, bare bioanode, and encapsulated bioanode, respectively. In addition, the encapsulated alginate bioanode led to the highest reduction currents of (4.14 A·m^−2^) and HER of 0.39 m^3^·m^−3^·d^−1^. The relative bacterial distribution of *Geobacter* was 79%. The COD removal by all the bioanodes was between 62% and 88%. The findings of this study demonstrate that the MEC based on the encapsulated alginate bioanode exhibited notably higher bio-electroactivity compared to both bare, alginate bioanode, and an encapsulated bioanode. We hypothesize that this improvement in electron transfer rate is attributed to the preservation and the biofilm on the anode material using alginate hydrogel which was inserted into a filter bag.

## 1. Introduction

Conventional wastewater treatment processes are energy-demanding, consuming 3–5% of the world’s electricity [1]. Therefore, it is essential to improve wastewater treatment efficiency by using new technologies that can harvest energy from the wastewater and convert it to fuels or electricity during mineralization. A bio-electrochemical system (BES), which includes microbial fuel cells (MFCs) for electricity generation and microbial electrolysis cells (MECs) for hydrogen production, offers significant economic, environmental, and technological advantages over traditional aerobic wastewater treatments [2,3,4,5]. In MECs, exoelectrogenic bacteria in the anode oxidize organic matter, leading to electron and proton generation. The electrons are transferred from the exoelectrogenic bacteria to the anode material, and then through an external wire to the cathode. At the same time, the protons are released into the solution and transfer to the cathode. In an anaerobic environment, a platinum (Pt) catalyst on the cathode reduced the protons to H_2_. The bio-electroactivity in MECs does not happen naturally; it requires an external voltage of >0.2 V [6]. The bio-electroactivity in BESs was reported to be influenced by temperature [7], pH [8], electrolyte solution (e.g., phosphate, acetate, and carbonate buffers) [9,10], microorganism species [11], organic substrates [12], anode-cathode spacing [13], and electrode materials [14]. We assume that of these factors, the anode is the most challenging component in MEC performance.

In order to increase the bio-electroactivity of the bacterial anode, attempts are made to improve the anode surface area and conductivity. For example, Truong et al. increased the anode conductivity by coating the anode (cellulose/polyaniline (BC/PANI)-based composites and chloride hexahydrate of iron (III) as oxidant (FeCl_3_·6H_2_O)) with titanium dioxide (TiO_2_). MFCs based on this anode led to a power density of 38.89 W·m^−3^, compared with 2.57 W·m^−3^ using a bare bacterial cellulose anode [15]. Yang et al. studied a three-dimensional anode made of cellulose-sponge carbon (made from wood pulp), obtained by carbonizing the wood pulp at temperatures of 600, 700, 800, 900, 1000, and 1100 °C. Carbonization at higher temperatures led to a lower charge transfer resistance. A multilayer porous surface of cellulose-sponge carbon was obtained when the carbonizing was performed at 1100 °C. This porous surface promotes microbial adhesion and thicker biofilms, with a biomass of 2661 ± 117 μg·cm^−2^, containing 86% electrogenic bacteria (*Geobacter*) and led to a maximum power density of 4.1 ± 0.1 W·m^−2^ [16].

Immobilization in microbial fermentation is an attractive technique designed to limit bacterial cells’ movement in an aqueous medium and protect them from harsh environmental conditions. Among the most widely used immobilization materials are agar, carrageenan, alginate, and agarose, owing to their low toxicity and high porosity [17]. Christwardana et al. increased the biocompatibility of an anode made of carbon felt using cellulose microfibrils and carrageenan (a gel-forming polysaccharide). An MFC adapted with this modified anode produced a power density of 70.98 mW∙m^−2^, higher than an MFC that used plain carbon felt. It was reported that the presence of carrageenan changed the carbon-felt properties from hydrophobic to hydrophilic [18].

Alginate is the most commonly used material in fermentation technologies. However, only a few studies reported its application in BESs. Mohebrad et al. evaluated the impact of bacterial immobilization in beads made of calcium alginate, to enhance lipase and protease production for dairy wastewater treatment in a microbial electrochemical system. At an induced current of 8.0 mA, the maximum production of protease and lipase was 47.89 and 193.81 IU·L^−1^, respectively. In addition, compared to the non-alginate reactor, the enzyme production time decreased from 12 to 4 h [19]. Neethu et al. investigated an MFC utilizing a stainless-steel mesh cage anode containing an immobilized inoculum in an alginate-bead matrix. In this MFC, the chemical oxygen demand (COD) removal efficiency and the coulombic efficiency were 91.6 ± 2.1% and 12.0 ± 0.5%, respectively. While an MFC based on an anode made of only stainless-steel mesh led to 69.3 ± 2.2% and coulombic efficiency of 9.6 ± 0.5% [20]. These studies introduced the promising potential for fabricating high-performance anodes using low-cost and sustainable materials.

Our research focused on improving the biofilm attachment to the carbon-cloth anode using alginate. In addition, the modified bioanode was encapsulated by a filter bag that served as a physical barrier, to overcome its low mechanical strength and alginate degradation by certain bacterial species in wastewater. Hydrogen production and COD removal by the MEC based on an encapsulated alginate bioanode (alginate bioanode encapsulated by a filter bag) were compared with three controls: MECs based on a bare bioanode (non-immobilized bioanode), an alginate bioanode, and an encapsulated bioanode (bioanode encapsulated by a filter bag).

## 2. Materials and Methods

### 2.1. Microbial Culture

*G. sulfurreducens* (DSMZ 12127, Braunschweig, Germany) was grown in *Geobacter* medium (50 mL) (N′ 826, DSMZ, Braunschweig, Germany) including acetate (10 mM) as the sole carbon source. The culture was incubated in bottles in an 80% N_2_: 20% CO_2_ atmosphere, and shaken (120 rpm) at 30 °C until the formation of prominent red bacterial aggregates [4]. Then, the supernatant was poured off, and the concentrated bacterial sediment was collected from all the bottles into one bottle. The concentrated suspension (0.8 OD 590 nm) was measured using a GENESYS 10S UV-Visible spectrophotometer (Thermo Scientific, Waltham, MA, USA).

### 2.2. Treatment of Carbon Textile Using Cold Low-Pressure Nitrogen Plasma

A plasma cleaner system (RF of 60 Hz and power of 18 W, Harrick PDC-32G2-, Ithaca, NY, USA) was used to treat the carbon-cloth anode with cold low-pressure nitrogen plasma (2 min at 0.3 torrs) [21]. The plasma-treated carbon textile was connected to titanium wires. To preserve the carbon cloth surface’s hydrophilic nature it was kept in demineralized water until use [22].

### 2.3. Preparation of Bacterial Biofilm on the Plasma-Treated Carbon-Cloth Anode

The preparation of the bacterial biofilm on the plasma-treated carbon-cloth anode was performed in a single chamber MEC (100 mL) including a platinum-coated carbon-cloth cathode, plasma-treated carbon cloth, and an Ag/AgCl reference electrode (3.0 M KCl, +199 mV vs. SHE; ALS Co., Ltd., Osaka, Japan). The MECs were filled with *Geobacter* medium (90 mL) and inoculated with 10% of *G. sulfurreducens* (0.8 OD 590 nm). The systems were sparged with nitrogen and connected to a potentiostat (MultiEmStat3+, PalmSens; CL Houten, Vleugelboot, The Netherlands) and maintained for two weeks at 35 °C under an applied voltage of 0.3 V vs. Ag/AgCl. The *Geobacter* medium was replaced every five days. After the acclimation period, the observed current in each of the 16 bioanodes was about ±0.32 mA·cm^−2^ (2 mA). At this stage, 12 bioanodes were used for further immobilization, and the remaining four (only carbon cloth with the attached biofilm) served as the bare bioanode controls.

### 2.4. Preparation Three Sets of Bioanodes

The first set of immobilized anodes included a pre-acclimatized bioanode (plasma-treated carbon cloth plus pre-acclimatized *G. sulfurreducens*) that was enclosed in a filter bag (40 × 130 mm with pore size of 25 µm; Dulytek, Seattle, WA, US), designated as the encapsulated bioanodes. For preparing the second set of bioanodes, sodium alginate (3%) was added to sterile hot distilled water (10 mL) followed by stirring (10 min). The pre-acclimatized bioanodes were immersed in the cool alginate solution (one minute), followed by inserting into 100 mL BaCl_2_ (0.1 M) for one hour to complete the gelatinating process under anaerobic conditions (N_2_ and CO_2_ gas). The alginate bioanodes were rinsed thoroughly with a sterile *Geobacter* medium to remove unnecessary particles. The second set was designated as alginate bioanodes. The third set included pre-acclimatized bioanodes immobilized using sodium alginate as mentioned above, but also encapsulated by the filter bag—designated as encapsulated alginate bioanodes. Each set of bioanodes contained four replicates.

### 2.5. MEC Setup

**Scheme 1:**
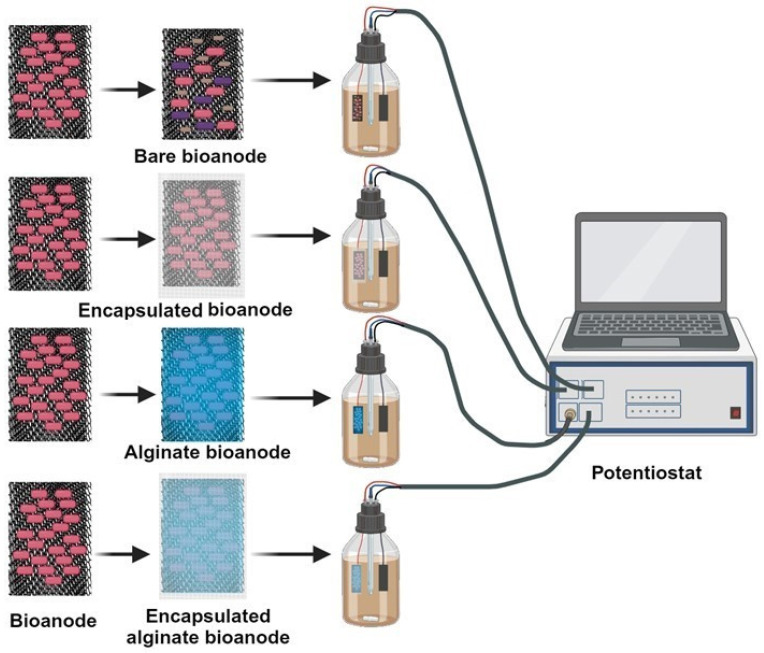
Bioanodes (Pre-acclimated bioanodes with *G. sulfurreducens*). Bare bioanode (without any modification), Encapsulated bioanode (bioanode inserted into a filter bag), Alginate bioanode (bioanode immersed with alginate), and Encapsulated alginate bioanode (bioanode immersed with alginate and inserted into a filter bag).

A single-chamber MEC was constructed using glass bottles (100 mL) (Isolab Laborgeräte GmbH, Eschau, Germany) containing a *Geobacter* medium (90 mL). The bottles were sealed with a silicone rubber septa stopper (GL-45, SCHOTT AG, Mainz, Germany) and a screw cap. The MEC included three electrodes: a carbon cloth cathode (2.5 cm × 2.5 cm) coated with platinum (Pt-0.5 mg·cm^−2^) (Cloth GDE, Fuel Cells Etc, Bryan, TX, USA), an Ag/AgCl reference electrode, and one of the bioanodes (the encapsulated bioanode, alginate bioanode, encapsulated alginate bioanode, and the bare bioanode). The cathode and the bioanode were separated by a polypropylene spacer net. The electrodes were connected to a potentiostat using titanium wires (Scheme 1). The MECs were filled with *Geobacter* medium (containing sodium acetate (10 mM)) or with cowshed wastewater (800 mg·L^−1^ COD) (the medium type is indicated in each experiment). The medium was replaced when there was a drop in the electrochemical activity, about once a week. Acetate was added twice a week. The MECs were placed on a magnetic stirrer and incubated at a temperature of 35 °C for 14–43 days as indicated for each experiment.

### 2.6. Bio-Electrochemical and Chemical Analysis

A MultiEmStat3+ potentiostat was connected to the MEC in a 3-electrode configuration and operated under 0.3 V vs. Ag/AgCl (3.0 M KCl). Linear sweep voltammetry (LSV) was performed in the potential range of −0.5 V to 0.8 V with a scan rate of 5 mV·s^−1^ [23,24]. Differential pulse voltammetry (DPV) was applied to the same potential range to determine the current–voltage (I–V) curve under a semi-steady state. Cyclic voltammetry (CV) and electrochemical impedance spectroscopy (EIS) experiments were conducted employing the potentiostat. The bioanode was designated as the working electrode, the cathode as the counter electrode, and the reference electrode was Ag/AgCl. The CV scans were performed at a rate of 50 mV·s^−1^ within the potential range of −0.8 V to 0.7 V. For EIS, a frequency range spanning 200 kHz to 0.01 Hz was employed, and the resulting EIS spectra were analyzed using ZView software (ZSimpWin 3.21). All the electrochemical analyses were performed after medium replacement when the systems showed stabilization in the electrochemical activity.

Chemical oxygen demand (COD) was determined using a colorimetric-based closed reflux COD digester (DBR-001, MRC, Holon, Israel).

The HER was measured in a full cell (2-electrode) configuration in the applied constant potential range of 0.2 to 0.8 V. The HER was calculated according to Equations (1) and (2) [6].

(1) $$Q_{H_{2}}=V_{H_{2}}\left[m^{3}\right]\times t\left[d^{-1}\right]\times V_{r}\left[m^{-3}\right]$$

Here, $V_{H_{2}}$ is the volume of hydrogen production (m^3^); t = time (seconds/day); V_r_ is the volume of the reactor in m^3^.

(2) $$V_{H_{2}}=\frac{I\times t\times R\times T}{k\times F\times P}$$

where I—current (A); t—time (s); R—gas constant (0.0820577 L·atm·mol^−1^· K^−1^); T—temperature (K); k—valence number of substrate; F—Faraday’s constant (96,485 C·mol^−1^); P—H_2_ gas pressure (atm).

### 2.7. Microbial Diversity Analysis

The microbial diversity of the different bioanodes was examined at the end of the experiment. The biofilm DNA was extracted using the DNeasy PowerSoil kit (Qiagen, Hilden, Germany) per the supplier’s guidelines. A 16s library preparation for sequencing on Illumina (San Diego, CA, USA) was performed using a 2-step PCR protocol. Sequencing was done on the Illumina Miseq, using a v2-500 cycles kit to generate 2 × 250 paired-end readings. Demultiplexing was performed on Basespace (the Illumina cloud) to generate FASTQ files for each sample. The data was further analyzed using CLC-bio to generate OTU and Abundance tables. The entire process was handled by Hylabs Ltd., Rehovot, Israel [25,26].

## 3. Results and Discussion

### 3.1. Cyclic Voltammetry Analysis and Nyquist Plots of the Different Bioanodes after 14 Days of Construction

**Figure 1:**
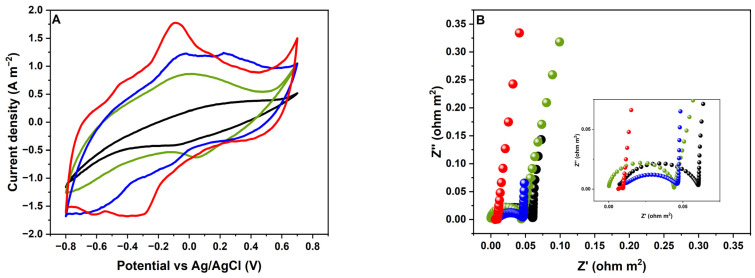
CV analysis (**A**) and Nyquist plots (**B**) on the 14th day of MECs fed with *Geobacter* medium based on the bare bioanode (black), encapsulated bioanode (green), alginate bioanode (blue), and encapsulated alginate bioanode (red).

MECs were constructed with anodes that were pre-acclimatized with *G. sulfurreducens* for 14 days until a stable biofilm was formed, followed by modification of the bioanodes with or without the alginate and the filter bag. The electrochemical behavior of MECs based on the encapsulated alginate bioanode was compared to MECs based on the immobilized bioanodes (encapsulated bioanode and alginate bioanode) and the non-immobilized (bare bioanode), while the MECs were fed with *Geobacter* medium for two weeks. CV and EIS measurements are shown in Figure 1A,B. The CV analyses of the MECs with the immobilized bioanodes showed significant redox peaks, indicating electrocatalytic activity (Figure 1A). The CV of the encapsulated alginate bioanode exhibited three redox peaks at potentials of −0.63 V, −0.45 V, and −0.09 V. The alginate bioanode exhibited redox peaks at −0.05 V and 0.22 V. The encapsulated bioanode showed one redox peak at −0.05 V. No peaks were observed for the bare bioanode.

The encapsulated alginate bioanode showed the highest exchange current density (1.78 A·m^−2^), represented by the Y axis. Meanwhile, the alginate bioanode and the encapsulated bioanode exhibited exchange current densities of 1.23 A·m^−2^ and 0.86 A·m^−2^, respectively. Immobilization of the biofilm on the anode with alginate and encapsulation with a filter bag led to significant differences in the shape of the voltammograms. In addition, the voltammetric response of the encapsulated alginate bioanode showed high Nernstian behavior. Furthermore, the encapsulated alginate bioanode exhibited a significant increase in oxidation and reduction in current peaks, which showed that immobilized biofilm on the encapsulated alginate bioanode enhanced electrocatalytic activity.

The internal resistance of the MECs with the immobilized bioanode was analyzed in a Nyquist plot from EIS data (Figure 1B). The charge transfer resistance (Rct) of the encapsulated alginate bioanode was 4.75 Ω, which was 17.8 times lower than that of the bare bioanode (84.82 Ω), 12.2 times lower than that of the alginate bioanode (57.86 Ω), and 14.5 times lower than that of the encapsulated bioanode (70.19 Ω). Low Rct indicates high electrochemical reaction efficiency and a higher electron transfer of the anode [27]. We assume that the immobilization and encapsulation of the biofilm enhanced the microorganisms’ adhesion and reduced the electron transfer resistance, as was shown in our previous studies [24,28].

### 3.2. CV Analysis and Nyquist Plots during 3 Weeks of MECs Based on the Encapsulated Alginate Bioanode

**Figure 2:**
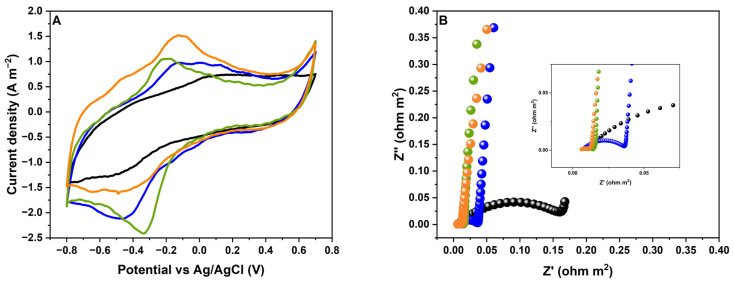
Cyclic voltammetry (**A**) and Nyquist plots (**B**) measurements of MECs based on the encapsulated alginate bioanode: time zero (black), after one week (blue), two weeks (green), and three weeks (orange).

As was shown in Figure 1, MECs occupying the encapsulated alginate bioanode exhibited lower resistance and higher oxidation-reduction peaks compared to the alginate bioanode, encapsulated bioanode, and bare bioanode. To figure out the bio-electroactivity development of the MEC based on the encapsulated alginate bioanode, CV and EIS were conducted for 3 weeks while the MECs were fed with *Geobacter* medium. The CV curves (Figure 2A) revealed a notably large peak at a potential of 0.13 V that increased over time from 0.3 to 0.95 mA. As depicted from the Nyquist plots of the anodes (Figure 2B), the ohmic values of the semi-circle diameter exhibited higher resistance Rct at the beginning of the experiment, 266 Ω, indicating a higher overpotential of the bioanodes in the absence of a mature biofilm. At the beginning of the operation, the Rct value for the encapsulated alginate bioanode was 240.2 Ω, which decreased over time and reached 49.6 Ω after one week of operation, eventually dropping to 9.8 Ω after three weeks of operation. We assume that the growth of the biofilm led to a drastic decrease in the Rct value. This finding may indicate that the developed biofilm greatly enhanced the electron transfer rate.

### 3.3. DPV and LSV Measurements of the MECs Based on the Encapsulated Alginate Bioanode Which Was Fed with Geobacter Medium and Wastewater at Different Ratios

A comparison of the current and hydrogen production in MECs based on the different bioanodes was performed while the MECs were fed with different ratios of *Geobacter* medium and wastewater. The measurements were performed on the 30th day, and on the 36th day of the MEC operation, when the MECs were fed with *Geobacter* medium and wastewater in different dilutions (2:1 and 1:1, respectively) (800 mg·L^−1^ COD); and on the 43rd day, when the MECs were fed with only wastewater (896 mg·L^−1^ COD). The bacterial bioanodes LSV oxidation was examined at 14 potentials between −0.5 to 0.8 V vs. Ag/AgCl, with a scan rate of 5 mV·s^−1^.

**Figure 3:**
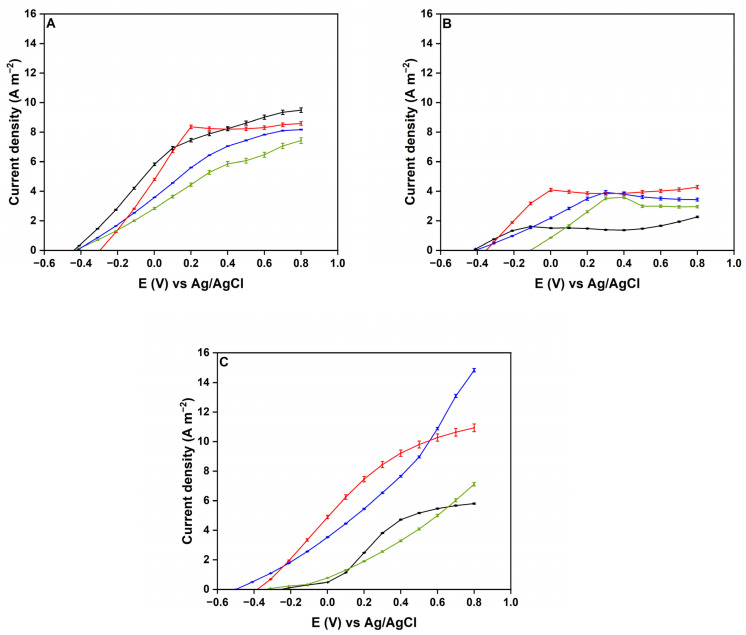
LSV oxidation measurements for MECs based on bare bioanode (black), encapsulated bioanode (green), alginate bioanode (blue), and encapsulated alginate bioanode (red), when the MECs were fed with *Geobacter* medium and wastewater in a single-chamber MEC. (**A**) On the 30th day with *Geobacter* medium and wastewater at the ratio of 2:1 (COD of 800 mg·L^−1^); (**B**) On the 36th day with *Geobacter* medium and wastewater at the ratio of 1:1 (COD of 800 mg·L^−1^); (**C**) On the 43rd day with only wastewater (COD of 896 mg·L^−1^).

LSV analyses (Figure 3A) were taken on the 30th day when the systems were fed with acetate and wastewater at a ratio of 2:1 (COD of 532 and 266 mg·L^−1^, respectively). The maximum current density of the bare bioanode was 9.49 ± 0.15 A·m^−2^, and that of the encapsulated alginate bioanode was 8.59 ± 0.12 A·m^−2^. The encapsulated bioanode and alginate bioanode led to a maximum current density of 7.42 ± 0.19 A·m^−2^ and 8.17 ± 0.026 A·m^−2^, respectively, at an applied potential of 0.8 V. LSV analyses on the 36th day (Figure 3B), when the MECs were fed with acetate and wastewater at the ratio of 1:1 (COD of 800 mg·L^−1^), showed that increasing the ratio of the wastewater resulted in a decrease in the current densities by all the bioanodes (Figure 3B). In addition, it was observed that all the bioanodes had a plateau curve from their potential of the highest current density up to 0.8 V. The encapsulated alginate bacterial bioanode obtained the highest current density at 0 V, which was 4.09 ± 0.11 A·m^−2^. In contrast, the bare bioanode anode reached its highest current density at 0.8 V, where it was only 2.26 ± 0.05 A·m^−2^. LSV analyses on the 43rd day (Figure 3C), when the MECs were supplied with only wastewater (COD of 896 mg·L^−1^), showed that at an applied potential of 0.8 V, the bare bioanode, encapsulated bioanode, alginate bioanode, and encapsulated alginate bioanode led to current density values of 5.80 ± 0.04, 7.12 ± 0.12, 14.82 ± 0.11, and 10.93 ± 0.026 A·m^−2^, respectively; but the plateau started from 0.4 V in the encapsulated alginate bioanode. In summary, the alginate bioanode led to the highest current density at an applied voltage of 0.8 V. But at a lower applied voltage of 0.4 V, the encapsulated alginate bioanode led to the highest current density of 9.21 ± 0.16 A·m^−2^, which was 20%, 95%, and 180% higher than the alginate bioanode, bare bioanode, and encapsulated bioanode, respectively.

In addition, the leveling of the curve was measured at high anodic potentials, typically above 0.4 V. The onset of the encapsulated alginate bioanode was −0.4 V, while for the bare bioanode it was −0.2 V. From the results in Figure 3C, it can be seen that when the MEC was fed with wastewater, higher electroactivity was obtained by the encapsulated alginate bioanode. We attribute this to the current increase in the accumulation of reactive mediators, as seen in voltammograms of the encapsulated alginate bioanode (Figure 1), as well as to the growth of active biofilm on the bioanode with time (Figure 2); both of these effects lowered the charge transfer resistances. New soluble redox mediators in wastewater may have enhanced the bioanode reaction, facilitating additional charge transfer at high potentials above the limiting current of 0.4 V.

### 3.4. LSV Reduction Currents of MECs Based on the Different Bioanodes Fed with Geobacter Medium and Wastewater at Different Ratios

The current density and hydrogen production in MECs based on the different bioanodes were examined while the MECs were fed with varying ratios of *Geobacter* medium and wastewater, as described for LSV oxidation currents (Figure 3). However, the measurements for LSV reduction currents were performed in a full cell (2-electrode configuration).

**Figure 4:**
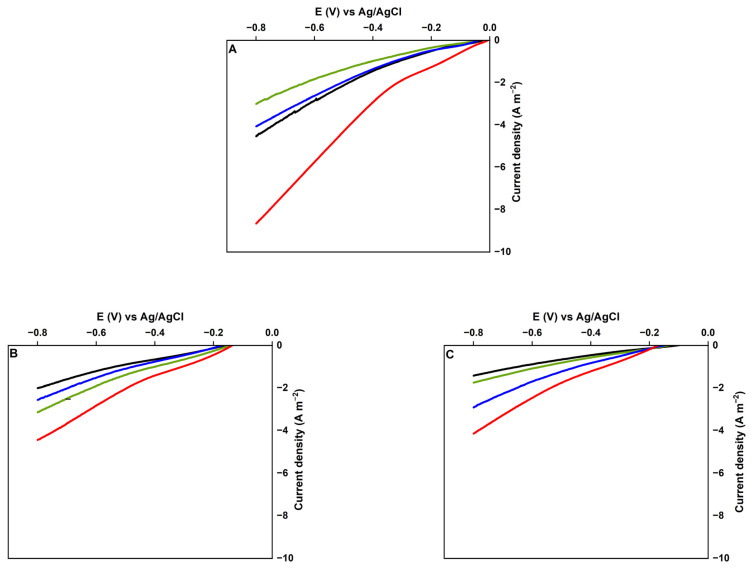
LSV reduction polarization curves for a cathode in single-cell MECs based on a bare bioanode (black), encapsulated bioanode (green), alginate bioanode (blue), and encapsulated alginate bioanode (red). On the 30th and 36th days, when the MECs were fed with *Geobacter* medium and wastewater at a 2:1 (**A**) and 1:1 (**B**) ratio. On the 43rd day, with only wastewater as a substrate (**C**).

LSV steady-state polarization was observed for the cathode in MECs which were fed with *Geobacter* medium and wastewater. The results depicted in Figure 4A–C showed that the highest reduction current density, at an applied cell voltage of 0.8 V, was obtained in the MECs with the encapsulated alginate bioanode: 8.66 A·m^−2^, and 4.44 A·m^−2^, when the MECs were fed with *Geobacter* medium and wastewater at 2:1 and 1:1, respectively. When the MEC was fed with only wastewater, it was 4.14 A·m^−2^. The bare bioanode led to 4.53 A·m^−2^, 2.01 A·m^−2^, and 1.42 A·m^−2^ for the various ratios. In comparing the reduction current density of all bioanodes at an applied cell voltage of 0.8 V, when the MECs were fed with only wastewater, the currents of the bare bioanode, encapsulated bioanode, and alginate bioanode led to 1.42 A·m^−2^, 1.75 A·m^−2^, and 2.91 A·m^−2^, respectively. However, the encapsulated alginate bioanode led to the highest current, 4.14 A·m^−2^.

The HER was measured in the MECs using the different bioanodes as they were fed with *Geobacter* medium and wastewater at different ratios (Table 1). The MEC with the encapsulated alginate bioanode led to 0.82, 0.42, and 0.39 m^3^·m^−3^·d^−1^ when the MECs were fed with *Geobacter* medium and wastewater at ratios of 2:1; 1:1 and only wastewater, respectively. Meanwhile, the bare bioanode led to only 0.43, 0.19, and 0.13 m^3^·m^−3^·d^−1^, in these respective conditions.

Several studies showed that utilizing alginate to modify the anode in MFCs and MECs improved their bio-electroactivity (a comparison of the following electroactivity of MFC/MEC based on an anode modified with alginate is presented in Table 2). Neethu et al. showed that an MFC with an anode made of an alginate-bead matrix, mixed with activated carbon and anaerobic sludge in a stainless-steel mesh cage, showed a maximum power density of 2.6 W·m^−3^, compared to a similar MFC with a bare carbon felt (without the alginate) and stainless-steel mesh as an anode material, which led to a maximum power density of only 2.1 ± 0.3 W·m^−3^ [20]. Szollosi et al. studied an anode containing bacteria entrapped in alginate/polyaniline/TiO_2_/graphite gel. They observed that the addition of 0.05 g·mL^−1^ graphite powder resulted in a 105-fold increase in current and a 7-fold higher power density (7.88 W·m^−3^), compared to control experiments [29]. Zhao et al. developed a double-layer hydrogel anode based on sodium alginate. The inner hydrogel layer of encapsulated Fe_3_O_4_ and electroactive microorganisms was used as the bio-electrochemical catalytic layer. This MFC led to an open-circuit voltage of 1.17 V and an operating voltage of 781 mV [30]. Wang et al. improved the biocompatibility of the anode in an MFC by preparing a polyaniline/sodium alginate/carbon brush (PANI-SA/CB) anode. The easily fabricated PANI–SA-conducting hydrogels showed a maximum power density of 515 mW·m^2^, which was 1.38 times higher than that of the blank CB anode (373 mW·m^−2^). The researchers reported that the excellent capacitive properties of PANI-SA can function as a bio-capacitor, as it can simultaneously store electrons generated from microbial oxidation of substrates and release the accumulated charge [31]. Yong et al. developed an anode of immobilized electrogenic microorganisms using graphite/alginate granules. This MFC led to a much more stable electricity output than that with suspended microorganisms. It was reported that the coulombic efficiency was ∼0.8 to 1.7 times higher than the suspension mode. In addition, with the conductive graphite/alginate/cells granules, the internal resistance of the MFC decreased dramatically [32].

It is important to note that alginate has some disadvantages associated with its use for bacterial immobilization, including structural degradation, mass transfer limitations, low mechanical strength, and cell release [33]. Hubenova et al. entrapped the Gram-positive *Paenibacillus profundus* bacterium in an alginate polymer bonded onto graphite paper. An MFC with this anode led to a current density of 30 mA·m^−2^ at an applied potential of −200 mV (vs. SHE). This value was increased five-fold when artificial redox-active mediators (the redox dyes thiazolyl blue formazan and phenazine methosulfate) were incorporated into the polymer [34]. To optimize bacterial immobilization efficiency and avoid the release of cells from the support, some chemical methods have been proposed, like chitosan and glutaraldehyde. In our previous article, the anode in MEC was based on *G. sulfurreducens* immobilized with alginate and chitosan in a procedure that included a bacterial suspension (1.0 OD) with alginate (3% *w*/*v*); and after that, chitosan (0.25%) was added for improving the alginate polymerization. The alginate/chitosan anode led to a HER rate of 0.56 m^3^·m^−3^·d^−1^ at 0.5 V with only wastewater [24]. However, chitosan dissolves only in acidic solutions, which may influence the viability of *G. sulfurreducens*.

**Table 2:** Summary of literature review on bioelectrochemical systems based on anode modification using alginate.

Type of Reactor	Anode Modification	Type of Substrate	Type of Inoculum	Cell Volume (mL)	Improvement of the Bio-Electroactivity (Fold Higher) **	References
MFC	Gel-entrapped bacteria in alginate/polyaniline/TiO_2_/graphite composites	Modified LB medium	*Shewanella algae*	12	7	[30]
MFC	Double-layer sodium alginate hydrogel bioanodes	High-salinity waste leachate	Culture of electroactive microorganisms	250	3.3	[31]
MFC	Polymerization of a polyaniline/sodium alginate	Sodium acetate	Mixture of microorganisms	100	1.38	[32]
MFC	Immobilized electrogenic microorganisms using graphite/alginate granules	Electrolyte (M9 minimal medium LB medium, lactate)	*Shewanella oneidensis*	150	1.8	[33]
MFC	Alginate polymer bonded onto graphite paper	Peptone Meat Broth	*Paenibacillus profundus*	20	5	[35]
MFC	Stainless-steel mesh cage anode containing an alginate-bead matrix with immobilized inoculum	Synthetic wastewater	Anaerobic sludge inoculum	160	3.6	[20]
MEC	Immobilized carbon cloth with alginate and chitosan	Wastewater	*Geobacter sulffureducens* and wastewater	80	3.5	[24]
MEC	Encapsulated alginate bioanode	Wastewater	*Geobacter sulffureducens* and wastewater	90	3	Current study

** The bio-electroactivity improvement was assessed by comparing the modified anode with the control anode using power density in MFC systems and HER in MECs.

This current study focused on physical methods, using a filter bag to protect the immobilized bacteria in the alginate polymer on the carbon-cloth anode, in an attempt to overcome the challenge of alginate’s low mechanical strength.

### 3.5. Bacterial Diversity Analysis of the Different Bioanodes at the End of the MECs’ Operation

**Figure 5:**
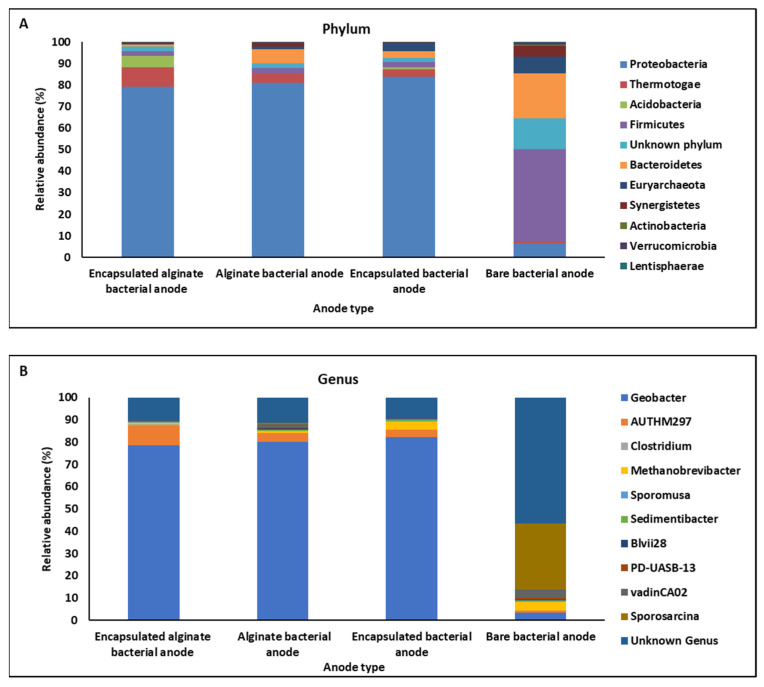
Relative bacterial distribution by phylum (**A**) and genus (**B**) in the biofilm of the different bioanodes in MECs fed with wastewater.

Examination of bacterial diversity analysis of the different bioanodes was performed at the end of the MECs’ operation. The filter bag was removed from the encapsulated bioanodes (encapsulated bioanode and encapsulated alginate bioanode). Then, all the different bioanodes were gently washed to remove planktonic bacteria. The relative bacterial distribution in the biofilms on the anodes was analyzed based on the 16S rRNA. As depicted in Figure 5, the most abundant phyla of the different immobilized bacterial anodes (encapsulated bioanode, alginate bioanode, and encapsulated alginate bioanode) were the *Proteobacteria* with relative distributions ranging from 79%, 81%, and 84%, respectively. However, in the bare bacterial bioanode, the relative distribution was only 6%. The remaining phyla (*Firmicutes, Thermotogae*, *Acidobacteria*, etc.) were about 15% in the immobilized bioanodes. In the MEC with the bare bioanode, the dominant phyla were *Firmicutes* (43%) and *Bacteroidetes* (21%).

The proportion of *Geobacter* genera in the MEC reactors with immobilized bioanodes was around 79–82%. In contrast, the bare bioanode included only 3% *Geobacter*. Another genus was AUTHM297 (3–9%), and an unknown genus (9–11%) was observed in the immobilized bioanodes. In the case of the bare bioanode, an unknown genus (56%), *Sporosarcina* (30%), and *Methanobrevibacter* (4%) were observed. In summary, the immobilized bioanodes included a significantly higher relative distribution of the exoelectrogenic bacteria *Geobacter* than the bare anode.

The dominant phylum in MECs and MFCs is mostly *Proteobacteria*, which are known for electrochemical activity. Bacteria in the *Proteobacteria* phylum are known for their ability to direct electron transfer to the anode material [35]. *Firmicutes* are known for their tolerance to harsh conditions. However, *Firmicutes* show relatively lower electrochemical activity, because they have thick cell walls, and electrons need to pass through the cell wall to the anode. *Clostridium* is a successful isolate of *Firmicutes* that has been applied in MFCs [36]. The remaining phylum, *Bacteroidetes*, is commonly identified in anaerobic digesters [37].

Studies showed that the dominant genus in MECs and MFCs is the *Geobacter* species [38]. Oshiai et al. evaluated the bio-augmentation of MECs with the *Geobacter sulfurreducens* strain YM18, and its effect of on current generation and hydrogen production. They found that the bio-augmented MEC generated a threefold increase in current during one month of operation and produced sixfold more hydrogen compared to the non-bio-augmented control. The successful colonization of anode surfaces with YM18 was affirmed through quantitative PCR and meta-barcoding analyses [39]. The *Methanobrevibacter* strain was observed in all four reactors. *Methanobrevibacter* is mainly present in domestic wastewater because of human waste. *Methanobrevibacter* can convert hydrogen gas into methane gas by reducing CO_2_ [40,41]. This study indicated that the growth of *Methanobrevibacter* with the alginate-encapsulated bioanodes was well inhibited, resulting in lower CH_4_ production and higher H_2_ production.

The relatively low bacterial distribution of *Geobacter* may explain the low H_2_ production in the MEC with the bare bioanode, compared to the immobilized bioanodes.

### 3.6. COD Removal in the MECs Based on the Different Bioanodes When Fed with Geobacter Medium and Wastewater at Different Ratios

**Figure 6:**
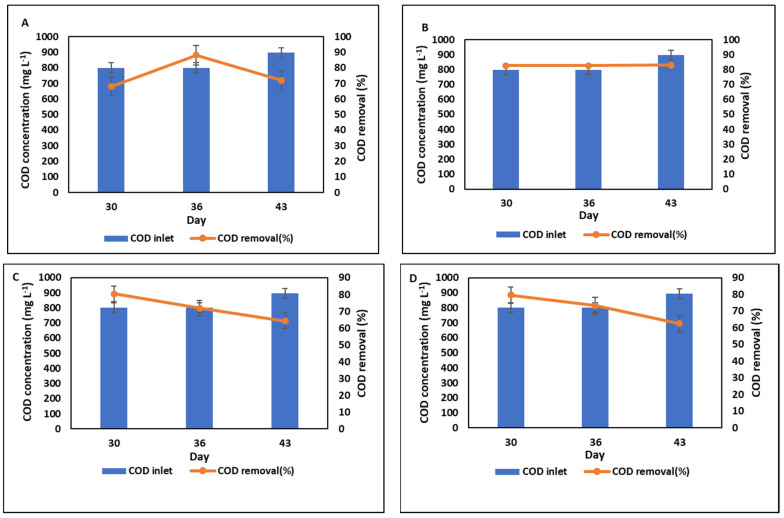
COD concentration (mg·L^−1^) and COD removal (%) in MECs based on the bare bioanode (**A**), encapsulated bioanode (**B**), alginate bioanode (**C**), and encapsulated alginate bioanode (**D**), at different days and ratios of *Geobacter* medium/wastewater. Day 30—2:1 ratio; Day 36—1:1 ratio; Day 43—0:1 ratio.

COD removal in the MECs occupying the different bioanodes was evaluated on the 30th, 36th, and 43rd days of the experiment, when *Geobacter* medium and wastewater were supplied at 2:1, 1:1, and 0:1 ratios, respectively (Figure 6). The COD removal by all the bioanodes was between 62% and 88%, regardless of the ratio of *Geobacter* medium and wastewater.

There are few reports of COD removal in MECs/MFCs that were based on bioanodes modified with alginate. Wang et al. constructed an MFC based on a polyaniline/sodium alginate/carbon-brush (PANI-SA/CB) anode and compared it to a blank CB bioanode. It was reported that the initial COD of all the MFC’s anode liquids was 520 mg·L^−1^. At the end of the cycle, the COD concentration of the MFC solution with the PANI-SA/CB hydrogel anode was 34 mg·L^−1^, which was 64% less than that in the blank CB anode (95 mg·L^−1^). The coulombic efficiency of the MFC equipped with the PANI-SA/CB hydrogel anode was 22% higher than that of the blank CB anode [31]. Neethu et al. examined COD removal in an MFC with an anode made of an alginate-bead matrix mixed with activated carbon and anaerobic sludge in a stainless-steel mesh cage. This MFC led to a COD removal efficiency of 91.6 ± 2.1%, but with bare carbon felt (without the alginate), the COD removal was only 69.3 ± 2.2%. However, the modified anode and the bare anode exhibited lower CE, less than 12% [20]. Hadiyanto et al. immobilized *Saccharomyces cerevisiae* yeast in alginate to utilize it as a micro-algae biocatalyst in the anode side of an MFC. Tofu wastewater was used as the substrate with an initial concentration of 1286 ± 21 mg·L^−1^. The alginate-immobilized anode (yeast concentration of 10.89% *w*/*v*) led to COD removal of 31.82%, while the suspended yeast in the anodic chamber of the control MFC system was 28.34% [42]. Gandu et al. constructed an MEC based on the immobilization of *Geobacter sulfurreducens* using alginate and chitosan and compared it with a non-immobilized anode. The COD removal percentage was about 75% in the MEC based on the immobilized anode, whereas in the MEC based on the non-immobilized anode, it was only 40% [24].

## 4. Conclusions

The current study focused on improving biofilm attachment to a carbon-cloth anode using an alginate hydrogel. In addition, the modified bioanode was encapsulated by a filter bag that served as a physical barrier, to overcome its low mechanical strength and alginate degradation by certain bacterial species in wastewater. An MEC based on an encapsulated alginate bioanode was compared with three controls: MECs based on a bare bioanode, alginate bioanode, and encapsulated bioanode.

During the time of operation, the encapsulated alginate bioanode led to a drastic decrease in the Rct value. This finding may indicate that the developed biofilm greatly enhanced the electron transfer rate. When the MECs were fed with only wastewater, the reduction currents of the bare bioanode, encapsulated bioanode, and alginate bioanode were 1.42 A·m^−2^, 1.75 A·m^−2^, and 2.91 A·m^−2^, respectively. The encapsulated alginate bioanode led to the highest currents (4.14 A·m^−2^). The MEC with the encapsulated alginate bioanode led to 0.82, 0.42, and 0.39 m^3^·m^−3^·d^−1^ when the MECs were fed with *Geobacter* medium and wastewater at ratios of 2:1, 1:1, and only wastewater, respectively. At the end of the MEC operation, the relative bacterial distribution on the encapsulated alginate bioanode showed that the genus *Geobacter* was 79%, while the bare bioanode led to only 3%. The COD evaluation of all bioanodes showed a COD removal of between 62% and 88%, regardless of the ratio of *Geobacter* medium and wastewater. The study showed the efficiency of an encapsulated alginate bioanode as an anode in the operation of MECs, a step towards achieving increased bio-hydrogen production along with wastewater treatment.

## Figures and Tables

**Table 1:** HER (at 0.8 V) when the MECs were fed with *Geobacter* medium/wastewater at different ratios.

**Geobacter** Medium: Wastewater	(2:1)		(1:1)		(0:1)	
Anode type in MEC	mA	H_2_ (m^3^·m^−3^·d^−1^)	mA	H_2_ (m^3^·m^−3^·d^−1^)	mA	H_2_ (m^3^·m^−3^·d^−1^)
Bare bioanode	2.83	0.43	1.25	0.19	0.88	0.13
Encapsulated bioanode	1.87	0.28	1.96	0.30	1.09	0.16
Alginate anode	2.53	0.38	1.59	0.24	1.82	0.28
Encapsulated alginate bioanode	5.41	0.82	2.77	0.42	2.58	0.39

## Data Availability

The original contributions presented in the study are included in the article; further inquiries can be directed to the corresponding author.
